# Association of early serial serum NLRP3 measurements with 28-day mortality in sepsis: a single-center retrospective landmark cohort study

**DOI:** 10.1186/s12879-026-13650-7

**Published:** 2026-06-01

**Authors:** Xiaoqin Lai, Junsheng Wang, Yujing Sun, Jueying Lin, Lin Wang, Zanxi Fang, Kuncheng Chen

**Affiliations:** 1 Department of Intensive Care Unit, Zhongshan Hospital Xiamen University, School of Medicine, Xiamen University, Xiamen, 361005 China; 2 Center of Clinical Laboratory, Zhongshan Hospital Xiamen University, School of Medicine, Xiamen University, Xiamen, 361005 China

**Keywords:** Sepsis, Serum NLRP3, Serial measurement, Biomarker, Prognosis

## Abstract

**Background:**

Sepsis is a heterogeneous, time-dependent syndrome in which static biomarkers may inadequately capture evolving host responses. We investigated whether baseline and early serial serum NLR family pyrin domain-containing 3 (NLRP3) measurements were associated with disease severity and 28-day mortality in sepsis.

**Methods:**

Adult ICU patients with sepsis were retrospectively enrolled. Serum NLRP3 was measured on ICU days 1 and 3. Early change was defined as ΔNLRP3 (day 3 minus day 1). Analyses involving ΔNLRP3 used a prespecified day-3 landmark design. The primary complete-case logistic model adjusted for age, sex, admission SOFA score, and log-transformed day-1 lactate. Multiple imputation addressed missing day-1 lactate values, and bootstrap resampling was used for internal validation.

**Results:**

The day-3 landmark cohort included 161 patients; 33 died by day 28. Baseline NLRP3 correlated weakly with admission SOFA score (Spearman’s ρ = 0.25, *P* = 0.0013) and showed limited standalone discrimination for post-landmark 28-day mortality (AUC, 0.605; 95% CI, 0.496–0.713). In the complete-case cohort (*n* = 147), higher ΔNLRP3 was independently associated with mortality, with an adjusted odds ratio (aOR) of 2.03 per 1-ng/mL increase (95% CI, 1.49–2.93; *P* < 0.001). This association remained consistent after multiple imputation (pooled aOR, 2.15; 95% CI, 1.53–3.02; *P* < 0.001). Adding ΔNLRP3 to the baseline clinical model improved the AUC from 0.669 to 0.828, with an optimism-corrected AUC of 0.802 after bootstrap validation. Day-3 NLRP3 had similar standalone discrimination to ΔNLRP3 (AUC, 0.788 vs. 0.796; DeLong *P* = 0.873).

**Conclusions:**

Early serial serum NLRP3 assessment was associated with post-landmark 28-day mortality and provided incremental prognostic information beyond admission clinical severity. The comparable exploratory performance of day-3 NLRP3 suggests that a single day-3 reassessment warrants further validation as a pragmatic risk-updating approach.

**Supplementary Information:**

The online version contains supplementary material available at 10.1186/s12879-026-13650-7.

## Introduction

Sepsis remains a leading cause of mortality and critical illness worldwide despite advances in antimicrobial therapy, organ support, and systems-level quality improvement. Contemporary epidemiological estimates continue to show a substantial global burden of sepsis-related incidence and death, underscoring the ongoing need for improved risk stratification and prognostic assessment in critical care [1]. Clinically, sepsis is defined as life-threatening organ dysfunction caused by a dysregulated host response to infection, operationalized by an acute increase in Sequential Organ Failure Assessment (SOFA) score of at least 2 points [2, 3]. This framework highlights that outcomes in sepsis are shaped not only by pathogen burden, but also by the evolving host response over time.

Current sepsis management guidelines strongly emphasize early recognition and timely treatment, yet accurate early prognostic assessment remains challenging [4]. Although severity scores such as SOFA are clinically useful, they primarily capture downstream physiological derangements and do not directly quantify the upstream immune processes that may determine whether a patient is moving toward recovery or persistent dysregulation. Sepsis is increasingly understood as a dynamic and heterogeneous syndrome characterized by overlapping inflammatory and anti-inflammatory programs, immune cell dysfunction, and variable degrees of immunosuppression that may emerge early and persist over time [5–8]. This biological heterogeneity likely contributes to the inconsistent performance of many single time-point biomarkers.

Accordingly, sepsis biomarker research has increasingly shifted toward serial and trajectory-based assessments rather than isolated baseline measurements. A pragmatic parallel already exists in routine clinical practice, where repeated lactate measurements and lactate dynamics are used to assess response to resuscitation and to inform prognosis [4, 9]. Similar kinetic approaches have been explored for other sepsis biomarkers, including delta-procalcitonin [10]. More broadly, biomarker-development frameworks emphasize that clinically useful biomarkers should be evaluated with explicit attention to timing, context of use, and intended clinical decision-making [11, 12]. In this setting, early within-patient biomarker changes may provide information that is not captured by a single admission value alone.

The NLRP3 inflammasome is a central innate immune signaling platform that regulates caspase-1 activation and the maturation of interleukin-1β (IL-1β) and interleukin-18 (IL-18), thereby linking pathogen- and danger-associated signals to inflammatory amplification and pyroptotic pathways [13, 14]. Experimental studies have implicated NLRP3 signaling in organ injury and survival in sepsis [15]. Translational and integrative evidence further suggests that inflammasome-related pathways intersect with oxidative stress, endothelial injury, immunothrombosis, and later immune dysregulation, providing a biologically plausible link between early innate immune activation and downstream organ dysfunction [16–18].

Clinical studies have suggested that serum NLRP3-related measures are feasible in critically ill patients and may be associated with disease severity and prognosis. Serum NLRP3 has been linked to septic shock risk and short-term mortality [19], and has also been studied in specific sepsis phenotypes such as sepsis-associated acute respiratory distress syndrome [20]. In addition, sequential changes in NLRP3 inflammasome activation have previously been associated with mortality in sepsis [21], and inflammasome-related activation signatures have also been explored in vivo [22]. However, clinical evidence specifically evaluating early serial serum NLRP3 concentrations with an explicit landmark-based analytic framework remains limited. As sepsis biology and treatment response evolve rapidly during the first ICU days, reliance on a single time-point may fail to capture clinically relevant changes in host-response status.

A major methodological challenge in evaluating early biomarker kinetics is guarantee-time bias, which arises when a biomarker is defined using information obtained after time zero, while outcomes are counted from admission [23, 24]. Landmark analysis is a recognized approach to mitigate this bias by redefining follow-up from a prespecified landmark time and restricting the analytic cohort to individuals who remain at risk at that time [23, 25]. This issue is particularly relevant for change-based biomarkers such as ΔNLRP3, because calculation of the change inherently requires survival and measurement availability through the later time-point.

In this context, we conducted a retrospective observational cohort study of adult ICU patients with sepsis, measuring serum NLRP3 on ICU days 1 and day 3 and defining the early change as ΔNLRP3 (day 3 minus day 1). Using a prespecified day-3 landmark framework, we evaluated (i) the relationship between baseline serum NLRP3 and admission disease severity, and (ii) the association of early serial serum NLRP3 assessment with 28-day mortality. We further assessed whether ΔNLRP3 provided incremental prognostic discrimination beyond established admission clinical predictors. We hypothesized that baseline serum NLRP3 would primarily reflect contemporaneous illness severity, whereas early serial serum NLRP3 assessment may provide additional prognostic information for patients who remain in the ICU through day 3.

## Methods

### Study design and patient population

This retrospective observational cohort study was conducted in the intensive care unit (ICU) of a single tertiary hospital. Adult patients with sepsis admitted to the ICU between January 2022 and December 2023 were screened for eligibility. Sepsis was defined according to the Third International Consensus Definitions for Sepsis and Septic Shock (Sepsis-3). Patients were eligible if they fulfilled Sepsis-3 criteria and had a Sequential Organ Failure Assessment (SOFA) score of ≥ 2 at ICU admission, consistent with sepsis-related organ dysfunction.

Patients were excluded if they were younger than 18 years, had incomplete clinical data, or lacked the required paired serum NLRP3 measurements on ICU days 1 and 3; this latter category included patients who died before day 3 or were discharged from the ICU before the second sampling time-point. The primary endpoint was 28-day all-cause mortality.

### Landmark analysis

Because ΔNLRP3 was defined using measurements obtained on ICU days 1 and 3, analyses involving ΔNLRP3 were susceptible to guarantee-time bias if follow-up was counted from ICU admission. To mitigate this bias, a prespecified day-3 landmark design was used.

Only patients who were alive on day 3 and had serum NLRP3 measurements available on both day 1 and day 3 were included in the landmark cohort. For analyses involving ΔNLRP3, follow-up was defined from the day-3 landmark until death or day 28, whichever occurred first. ΔNLRP3 was treated as a covariate defined at the landmark time-point.

### Data collection and clinical variables

Demographic characteristics, comorbidities, infection site, microbiological data, organ-support therapies, and routine laboratory data were extracted from the electronic medical records. Disease severity was assessed using the SOFA score calculated at ICU admission (day 1). Clinical outcomes, including survival status at 28 days, were recorded for all patients. Organ-support therapies of interest included mechanical ventilation (MV) and continuous renal replacement therapy (CRRT). Routine laboratory variables included C-reactive protein (CRP), procalcitonin (PCT), and lactate measured on day 1. Microbiological variables, including culture status and Gram type, were recorded as baseline descriptors of the index sepsis episode. Secondary infection, nosocomial infection, or nosocomial culture positivity was not analyzed as a study outcome.

### Serum NLRP3 measurement

Serum samples were collected on ICU days 1 and 3. Day 3 was specifically selected because the first 48 to 72 h represent a critical clinical window for evaluating the initial response to sepsis resuscitation and empirical antimicrobial therapy [5, 26, 27]. Serum NLRP3 concentrations were measured using a commercially available Human NLRP3/NALP3 sandwich ELISA Kit (Colorimetric) (Catalog No. NBP3-18216, Novus Biologicals, Centennial, USA) according to the manufacturer’s instructions. The reported detection range was 0.31–20 ng/mL, with a sensitivity of 0.16 ng/mL. The reported intra-assay and inter-assay coefficients of variation were < 10% and < 15%, respectively. Because the manufacturer’s documentation does not specify whether the assay distinguishes full-length NLRP3 from circulating fragments or related NLRP3/NALP3 immunoreactive components, measured concentrations were interpreted as serum NLRP3-related protein levels rather than as a direct functional readout of intracellular inflammasome activity. Accordingly, measured circulating concentrations are described as serum NLRP3 or serum NLRP3-related protein levels throughout the manuscript, whereas “NLRP3 inflammasome” is reserved for discussion of the intracellular signaling pathway. Absorbance was measured at 450 nm using a Multiskan FC microplate reader (Thermo Fisher Scientific, Waltham, MA, USA).

Baseline serum NLRP3 was defined as the day-1 measurement. The early dynamic change in serum NLRP3 (ΔNLRP3) was defined as the day-3 concentration minus the day-1 concentration.

### Statistical analysis

Continuous variables were summarized as mean ± standard deviation or median (interquartile range), as appropriate, and categorical variables were presented as counts and percentages. Comparisons between groups were performed using Student’s t-test or the Mann–Whitney U test for continuous variables and the chi-square test or Fisher’s exact test for categorical variables, as appropriate.

The association between baseline serum NLRP3 and admission disease severity was evaluated using Spearman’s rank correlation analysis. The primary analysis for the association between ΔNLRP3 and 28-day mortality was performed in the prespecified day-3 landmark cohort using complete-case multivariable logistic regression. The primary multivariable model included age, sex, SOFA score at ICU admission, log-transformed day-1 lactate, and ΔNLRP3. To account for its right-skewed distribution, day-1 lactate was transformed using the natural logarithm as ln(1 + lactate) prior to inclusion in the regression models. To assess robustness, sensitivity models further adjusted for infection site, MV, or CRRT.

To compare the prognostic performance of serial NLRP3-related measures, standalone exploratory logistic regression models were fitted using day-1 NLRP3, day-3 NLRP3, or ΔNLRP3 as the only predictor. Predicted probabilities from logistic regression models were used to generate receiver operating characteristic (ROC) curves. The discriminative performance of the baseline clinical model, which included age, sex, admission SOFA score, and log-transformed day-1 lactate, was compared with that of extended models additionally incorporating ΔNLRP3 or day-3 NLRP3. Areas under the ROC curves (AUCs) were compared using DeLong’s test for correlated ROC curves. Improvement in model fit after adding ΔNLRP3 was assessed using the likelihood-ratio test. Model accuracy was additionally summarized using the Brier score for multivariable clinical prediction models.

Additional exploratory model comparisons were performed to assess whether day-1 NLRP3, day-3 NLRP3, and ΔNLRP3 provided overlapping or incremental information. First, a model including both baseline day-1 NLRP3 and ΔNLRP3 was compared with the primary ΔNLRP3 model using a likelihood-ratio test. Second, because the day-3 NLRP3 model and the ΔNLRP3 model are not nested, these models were compared using AUCs and DeLong’s test. For formal likelihood-ratio testing, reduced models containing one NLRP3-related measure were compared with a full two-time-point model containing both day-1 and day-3 NLRP3, in addition to the baseline clinical covariates.

Kaplan–Meier curves and log-rank testing were used descriptively to compare post-landmark survival between groups stratified by ΔNLRP3.

Primary multivariable, ROC, and exploratory model analyses were performed using complete-case data. Because day-1 lactate was missing in 14 patients (8.7%), a sensitivity analysis was performed using multiple imputation by chained equations under a missing-at-random assumption. Imputation was restricted to the prespecified day-3 landmark cohort and did not impute paired NLRP3 measurements for patients excluded before the landmark. Fifty imputed datasets were generated with 20 iterations using predictive mean matching for day-1 lactate. The imputation model included 28-day mortality, age, sex, admission SOFA score, day-1 lactate, day-1 NLRP3, ΔNLRP3, infection site, mechanical ventilation, continuous renal replacement therapy, comorbidity, culture status, Gram type, C-reactive protein, and procalcitonin. Lactate was imputed on the original scale and subsequently transformed as ln(1 + lactate) within each imputed dataset. The primary logistic regression model was fitted in each imputed dataset, and estimates were pooled using Rubin’s rules.

To assess the potential influence of overfitting and to internally validate model performance, bootstrap validation was performed using 1,000 resamples in the complete-case day-3 landmark cohort. In each bootstrap resample, the model was refitted and model performance was evaluated both in the bootstrap sample and in the original complete-case cohort. Optimism was estimated as the mean difference between bootstrap-sample and test-sample performance. Optimism-corrected AUCs and Brier scores were calculated for the baseline clinical model, the extended model including ΔNLRP3, and the extended model including day-3 NLRP3. Bootstrap percentile confidence intervals were also calculated for the ΔNLRP3 effect estimate.

All statistical analyses were conducted using R software (version 4.4.1; R Foundation for Statistical Computing, Vienna, Austria). All tests were two-sided, and *P* < 0.05 was considered statistically significant.

### Ethical approval

The study protocol was approved by the Institutional Ethics Committee of Zhongshan Hospital Xiamen University. No approval number was assigned. The study was conducted in accordance with relevant institutional guidelines and regulations and with the Declaration of Helsinki. Owing to the retrospective observational design and the use of de-identified clinical data and serum samples, the requirement for informed consent was waived by the ethics committee.

## Results

### Study population and sampling timeline

A total of 285 ICU patients with sepsis were screened for eligibility. After exclusions, 161 patients who were alive at day 3 and had serum NLRP3 measurements available on both day 1 and day 3 comprised the prespecified day-3 landmark cohort. All included patients met Sepsis-3 criteria and had a SOFA score of ≥ 2 at ICU admission. Serum samples were collected on ICU day 1 and day 3, and baseline clinical variables, including admission SOFA score, were recorded at ICU admission.

**Fig. 1:**
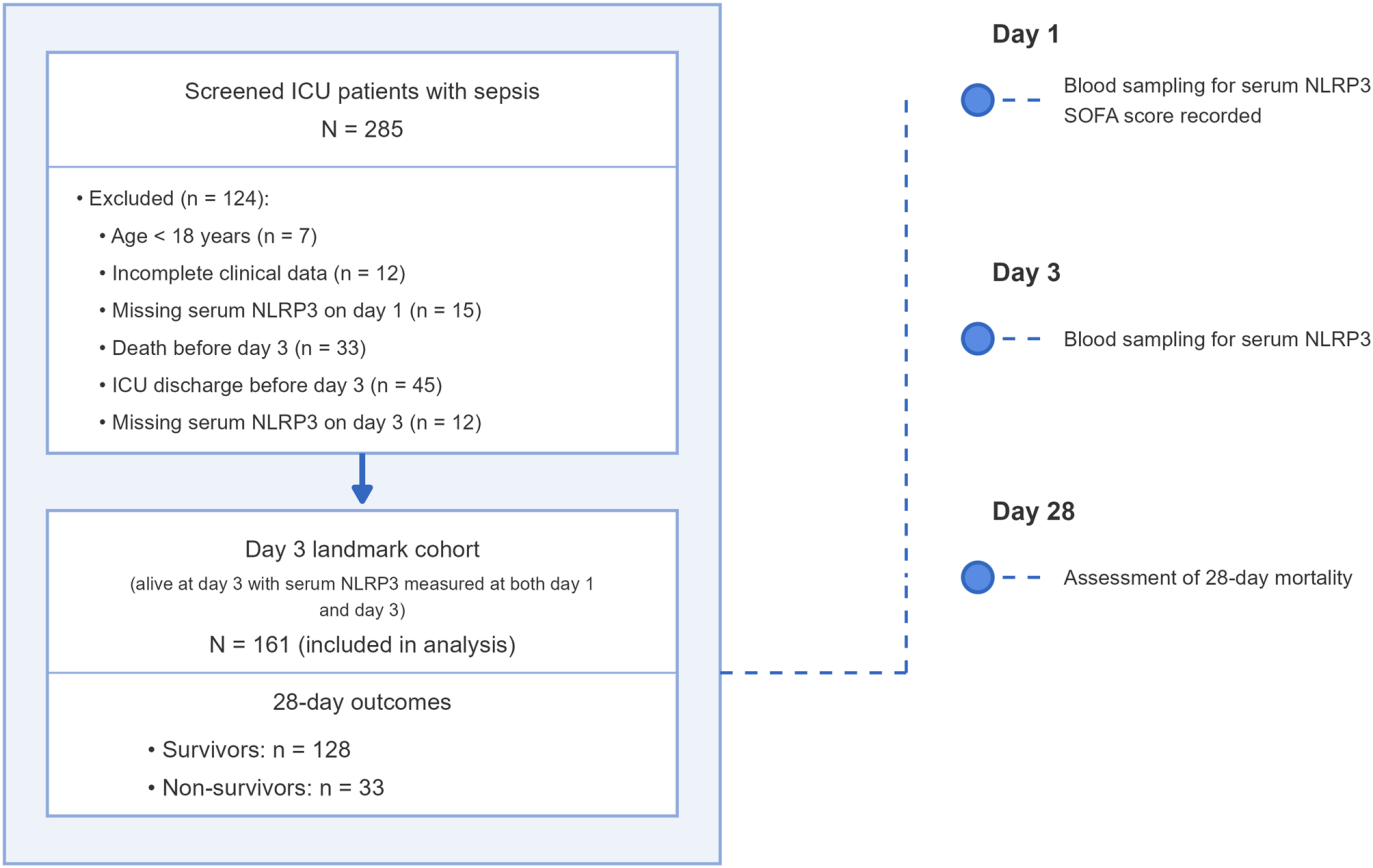
Study flow diagram and sampling timeline. ICU patients with sepsis were screened, and a prespecified day-3 landmark cohort was constructed including patients who were alive at day 3 and had serum NLRP3 measured on both day 1 and day 3. The primary endpoint was 28-day all-cause mortality

The primary endpoint was 28-day all-cause mortality. Among the 161 patients in the landmark cohort, 33 died by day 28. Because day-1 lactate was missing in 14 patients, the primary complete-case multivariable analyses included 147 patients, among whom 30 died after the day-3 landmark. The study flow diagram and sampling timeline are shown in Fig. 1.

### Baseline characteristics

The baseline characteristics of the landmark cohort are summarized in Table 1. The median age was 66 years, and the median SOFA score at ICU admission was 6. The lung was the most common infection site (42.2%), and 73.3% of patients had culture-positive infections. Median day-1 serum NLRP3, day-3 serum NLRP3, and ΔNLRP3 values for the landmark cohort are also shown in Table 1. Baseline characteristics stratified by 28-day survival status are provided in Additional file 1: Table S1.

**Table 1:** Baseline characteristics of the day-3 landmark cohort

Characteristic	Overall
Age, years	66.0 (53.0–79.0)
Sex, n (%)	
Female	75 (46.6)
Male	86 (53.4)
SOFA score at day 1	6.0 (4.0–8.0)
Infection site, n (%)	
Lung	68 (42.2)
Abdomen	37 (23.0)
Urinary tract	32 (19.9)
Other	24 (14.9)
Culture-positive, n (%)	118 (73.3)
Gram type, n (%)	
Gram-negative	62 (38.5)
Gram-positive	43 (26.7)
Other	14 (8.7)
Unknown	42 (26.1)
Any comorbidity, n (%)	93 (57.8)
Continuous renal replacement therapy, n (%)	51 (31.7)
Mechanical ventilation, n (%)	81 (50.3)
NLRP3 at day 1, ng/mL	2.98 (2.31–4.43)
NLRP3 at day 3, ng/mL	3.30 (1.42–4.01)
ΔNLRP3, ng/mL	-0.38 (-1.70–0.99)
C-reactive protein at day 1, mg/L	146.03 (95.21–197.99)
Procalcitonin at day 1, ng/mL	8.00 (3.99–15.19)
Lactate at day 1, mmol/L	3.85 (1.96–6.05)

Note. Continuous variables are presented as median (interquartile range), and categorical variables are presented as number (percentage). Day-1 lactate was available in 147 patients. ΔNLRP3 was defined as day-3 minus day-1 serum NLRP3 concentration. SOFA, Sequential organ failure assessment; CRRT, Continuous renal replacement therapy; NLRP3, NLR family pyrin domain-containing 3.  Because ΔNLRP3 was calculated within individuals, its median is not equal to the difference between the cross-sectional medians at day 3 and day 1

### Comparison according to availability of day-1 lactate

Among the 161 patients in the day-3 landmark cohort, day-1 lactate was missing in 14 patients (8.7%). Baseline characteristics were broadly similar between patients with and without available day-1 lactate (Additional file 1: Table S4). Post-landmark 28-day mortality was similar between the lactate-available and lactate-missing groups (20.4% vs. 21.4%; *P* = 1.000). Patients with missing day-1 lactate were less likely to receive mechanical ventilation (21.4% vs. 53.1%; *P* = 0.024), whereas age, admission SOFA score, infection site, culture status, day-1 NLRP3, day-3 NLRP3, and ΔNLRP3 did not differ significantly between groups.

### Association between baseline serum NLRP3 and disease severity

**Fig. 2:**
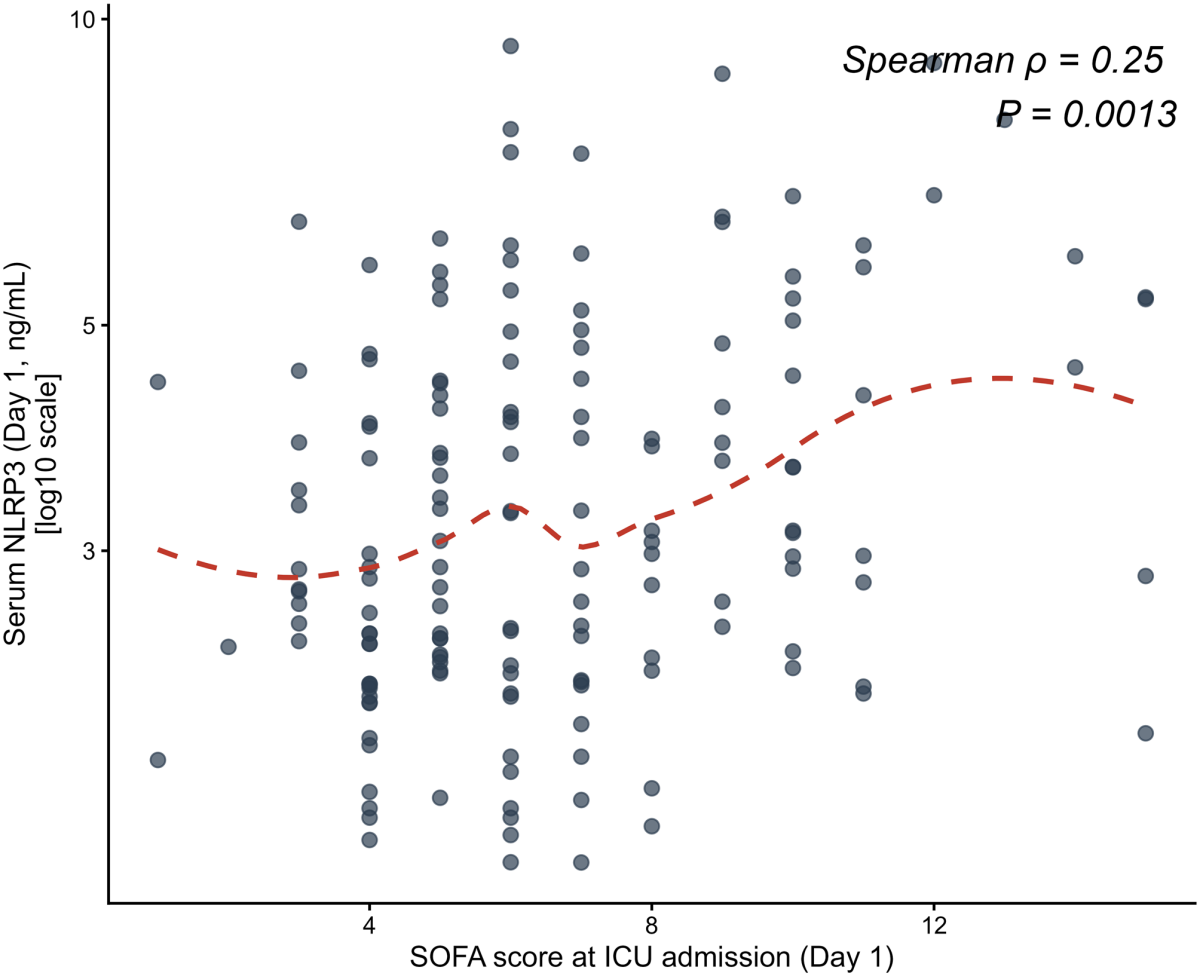
Association between baseline serum NLR family pyrin domain-containing 3 (NLRP3) levels and disease severity during intensive care unit (ICU) admission. Scatter plot showing the relationship between serum NLRP3 levels measured on day 1 and Sequential Organ Failure Assessment (SOFA) score at ICU admission among the study population (*n* = 161). The association was assessed using Spearman’s rank correlation analysis. A weak but statistically significant positive correlation was observed (Spearman’s ρ = 0.25, *P* = 0.0013). The dashed line represents a locally weighted smoothing curve to illustrate the overall trend. The y-axis is log10-transformed for visual clarity due to right-skewed extreme values

Baseline serum NLRP3 measured on day 1 was positively associated with admission disease severity. Spearman’s rank correlation analysis showed a weak but statistically significant correlation between baseline serum NLRP3 concentration and SOFA score at ICU admission (ρ = 0.25, *P* = 0.0013; Fig. 2), indicating that higher baseline serum NLRP3 levels were observed in patients with greater organ dysfunction at presentation.

### Association between ΔNLRP3 and post-landmark survival

**Fig. 3:**
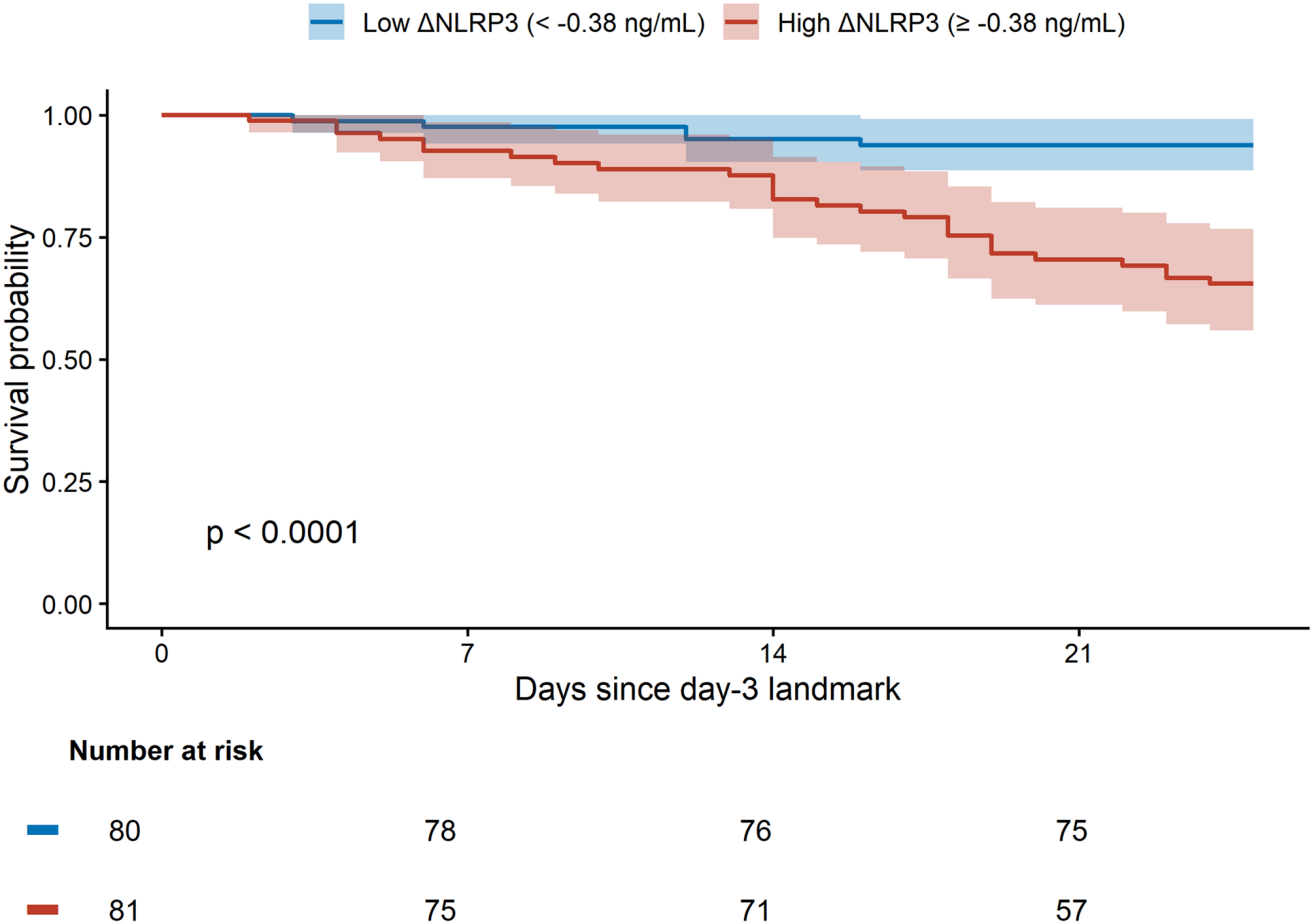
Kaplan–Meier analysis of post-landmark survival stratified by ΔNLRP3. Kaplan–Meier curves comparing post-landmark survival through day 28 between patients with low and high ΔNLRP3, dichotomized according to the cohort median ΔNLRP3 value of -0.38 ng/mL. Analyses were performed in the prespecified day-3 landmark cohort including patients who were alive at day 3 and had serum NLRP3 measured on both day 1 and day 3. Survival differences were assessed using the log-rank test

Kaplan–Meier analysis performed within the prespecified day-3 landmark cohort showed a significant association between ΔNLRP3 and post-landmark survival. Patients with higher ΔNLRP3 had significantly lower survival probabilities through day 28 than those with lower ΔNLRP3 (log-rank *P* < 0.0001; Fig. 3). These findings support an association between early increases in serum NLRP3 and worse subsequent outcomes after the day-3 landmark.

### Multivariable association of ΔNLRP3 with post-day-3 28-day mortality

In the primary complete-case multivariable logistic regression model adjusted for age, sex, admission SOFA score, and log-transformed day-1 lactate, higher ΔNLRP3 was independently associated with higher post-day-3 28-day mortality (aOR per 1-ng/mL increase, 2.03; 95% CI, 1.49–2.93; *P* < 0.001; Table 2). Admission SOFA score also remained associated with mortality (aOR, 1.24; 95% CI, 1.01–1.55; *P* = 0.045), whereas age, sex, and log-transformed day-1 lactate were not statistically significant.

**Table 2:** Multivariable logistic regression for post-day-3 28-day mortality in the day-3 landmark cohort

Variable	aOR	95% CI	*P* value
Age, per year	0.98	0.96–1.01	0.199
Male sex	1.11	0.43–2.96	0.829
SOFA score at day 1, per point	1.24	1.01–1.55	0.045
Log-transformed day-1 lactate	1.32	0.41–4.50	0.646
ΔNLRP3, per 1 ng/mL increase	2.03	1.49–2.93	< 0.001

Note. Primary complete-case multivariable logistic regression model for post-day-3 28-day mortality in the prespecified day-3 landmark cohort (*n* = 147; 30 deaths). ΔNLRP3 was defined as day-3 minus day-1 serum NLRP3 concentration. SOFA, Sequential Organ Failure Assessment

The association between ΔNLRP3 and mortality remained robust in sensitivity analyses that further adjusted for infection site (aOR, 2.10; 95% CI, 1.52–3.10; *P* < 0.001), mechanical ventilation (aOR, 2.05; 95% CI, 1.50–2.97; *P* < 0.001), or CRRT (aOR, 2.03; 95% CI, 1.48–2.95; *P* < 0.001) (Additional file 1: Table S2). The association was also consistent in the multiple-imputation sensitivity analysis including all 161 patients in the day-3 landmark cohort. After imputation of missing day-1 lactate values, ΔNLRP3 remained independently associated with post-day-3 28-day mortality in the pooled primary model (aOR per 1-ng/mL increase, 2.15; 95% CI, 1.53–3.02; *P* < 0.001; Additional file 1: Table S5). Admission SOFA score also remained associated with mortality (aOR, 1.25; 95% CI, 1.02–1.55; *P* = 0.035), whereas age, sex, and log-transformed day-1 lactate were not statistically significant.

In an exploratory model including both baseline day-1 NLRP3 and ΔNLRP3, model fit improved further compared with the ΔNLRP3 model alone (likelihood-ratio test *P* = 0.024) (Additional file 1: Table S3), suggesting that repeated NLRP3 assessment may carry more prognostic information than admission measurement alone.

### Discriminative performance of serum NLRP3-related measures

Day-1 serum NLRP3 alone showed limited standalone discrimination for post-day-3 28-day mortality (AUC, 0.605; 95% CI, 0.496–0.713). In contrast, ΔNLRP3 showed better standalone discrimination (AUC, 0.796; 95% CI, 0.689–0.902) and was significantly superior to day-1 NLRP3 (DeLong *P* < 0.001). Exploratory analysis showed that day-3 serum NLRP3 also had good standalone discrimination (AUC, 0.788; 95% CI, 0.701–0.875), with no significant difference compared with ΔNLRP3 (DeLong *P* = 0.873). These findings suggest that much of the prognostic signal may be captured by early day-3 reassessment, whereas admission NLRP3 alone had limited prognostic discrimination (Table 3).

**Table 3:** Discriminative performance of serum NLRP3-related measures and clinical models for post-day-3 28-day mortality

Model / Measure	AUC	95% CI	Brier score	Comparison	*P* value
Day-1 NLRP3 only	0.605	0.496–0.713	—	—	—
Day-3 NLRP3 only	0.788	0.701–0.875	—	vs. ΔNLRP3 only	0.873
ΔNLRP3 only	0.796	0.689–0.902	—	vs. Day-1 NLRP3 only	< 0.001
Baseline clinical model*	0.669	0.553–0.785	0.149	—	—
Baseline clinical model + ΔNLRP3	0.828	0.727–0.930	0.111	vs. baseline clinical model	0.004
Baseline clinical model + Day-3 NLRP3	0.814	0.725–0.903	0.123	vs. baseline clinical model	< 0.001

Baseline clinical model included age, sex, admission SOFA score, and log-transformed day-1 lactate. Brier scores are shown for multivariable clinical prediction models only; standalone biomarker-only logistic models are reported with AUC and 95% CI. All ROC analyses were performed in the complete-case day-3 landmark cohort (*n* = 147; 30 post-landmark deaths). P values for discrimination comparisons were calculated using DeLong’s test, where applicable. Direct comparison between the baseline clinical model + day-3 NLRP3 and the baseline clinical model + ΔNLRP3 showed no significant difference in discrimination (DeLong *P* = 0.712)

### Incremental prognostic value of ΔNLRP3 beyond the baseline clinical model

**Fig. 4:**
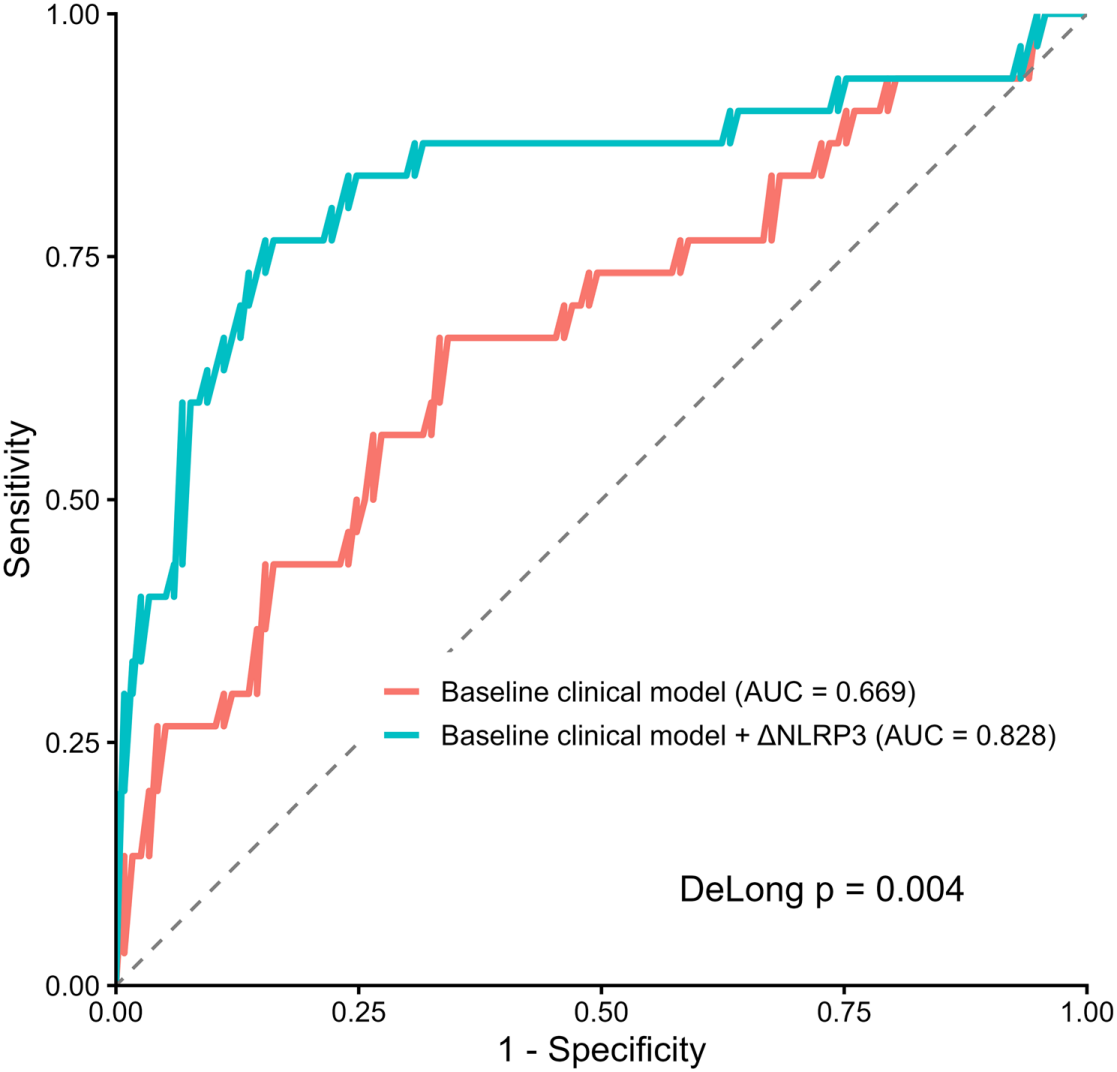
Incremental discriminative value of ΔNLRP3 beyond the baseline clinical model. Receiver operating characteristic curves comparing the baseline clinical model (age, sex, admission SOFA score, and log-transformed day-1 lactate) with an extended model additionally incorporating ΔNLRP3 for post-day-3 28-day mortality in the complete-case day-3 landmark cohort. Addition of ΔNLRP3 improved the AUC from 0.669 to 0.828

To evaluate whether ΔNLRP3 provided incremental prognostic information beyond established clinical predictors, we compared a baseline clinical model including age, sex, admission SOFA score, and log-transformed day-1 lactate with an extended model additionally incorporating ΔNLRP3. The extended model demonstrated significantly improved discrimination, with the AUC increasing from 0.669 (95% CI, 0.553–0.785) to 0.828 (95% CI, 0.727–0.930; DeLong *P* = 0.004; Fig. 4; Table 3). Consistently, addition of ΔNLRP3 significantly improved overall model fit (likelihood-ratio test χ²=24.90, *P* = 6.05 × 10^-7) and reduced the Brier score from 0.149 to 0.111 (Table 3). For comparison, a model incorporating day-3 NLRP3 instead of ΔNLRP3 also improved discrimination relative to the baseline clinical model (AUC, 0.814; 95% CI, 0.725–0.903; DeLong *P* < 0.001). Direct comparison of the day-3 NLRP3-extended model and the ΔNLRP3-extended model showed no significant difference in discrimination (AUC, 0.814 vs. 0.828; DeLong *P* = 0.712; Table 3). Additional exploratory model comparisons are shown in Additional file 1: Table S7. Compared with the baseline clinical model, adding day-1 NLRP3, day-3 NLRP3, ΔNLRP3, or both day-1 and day-3 NLRP3 significantly improved model fit. The full two-time-point model had the lowest AIC and Brier score (AIC, 122.73; Brier score, 0.108). In nested comparisons, the full two-time-point model improved fit compared with the day-3 NLRP3 model (LRT *P* = 0.0068) and the ΔNLRP3 model (LRT *P* = 0.0245), suggesting that day-1 and day-3 NLRP3 contain overlapping but not identical prognostic information. Together, these results indicate that early serial serum NLRP3 information may improve post-landmark risk stratification beyond admission clinical severity alone. Bootstrap internal validation showed that the incremental performance of the ΔNLRP3-extended model was not fully explained by apparent-sample optimism. The optimism-corrected AUC was 0.616 for the baseline clinical model and 0.802 for the baseline clinical model plus ΔNLRP3. The optimism-corrected Brier score was 0.161 for the baseline clinical model and 0.123 for the ΔNLRP3-extended model (Additional file 1: Table S6). The bootstrap median aOR for ΔNLRP3 was 2.11, with a bootstrap percentile 95% CI of 1.35–4.50. For comparison, the baseline clinical model plus day-3 NLRP3 had an optimism-corrected AUC of 0.781 and an optimism-corrected Brier score of 0.137.

## Discussion

In this single-center day-3 landmark cohort of ICU patients with sepsis, higher day-1-to-day-3 change in serum NLRP3 was independently associated with greater post-day-3 28-day mortality after adjustment for age, sex, admission SOFA score, and log-transformed day-1 lactate. Baseline serum NLRP3 showed a weak correlation with admission disease severity, whereas its standalone discriminative performance for post-landmark mortality was limited. In contrast, early serial serum NLRP3 information, summarized as ΔNLRP3, improved risk discrimination beyond the baseline clinical model. Taken together, these findings suggest that early serial serum NLRP3 assessment may provide clinically relevant risk-updating information in patients with sepsis who remain in the ICU through day 3.

These findings are consistent with the current view of sepsis as a dynamic and heterogeneous syndrome driven by a dysregulated host response rather than a static inflammatory state [2, 5–8]. In contemporary sepsis care, serial reassessment is central to clinical decision-making, and repeated measurements of biomarkers such as lactate are already used to evaluate early treatment response and ongoing physiological instability [4, 9]. Similar concepts have been explored for other biomarker kinetics, including delta-procalcitonin [10]. Within this broader framework, the present study supports the idea that repeated biomarker assessment may capture clinically relevant host-response evolution more effectively than a single admission measurement.

The biological plausibility of this observation is supported by the established role of the NLRP3 inflammasome in innate immune activation during sepsis. NLRP3 signaling is involved in caspase-1 activation and downstream maturation of IL-1β and IL-18, and has been linked to inflammatory amplification, organ injury, endothelial dysfunction, immunothrombosis, and later immune dysregulation in sepsis [13, 14]. More recent integrative reviews have further emphasized links between inflammasome-related pathways and oxidative stress, endothelial injury, immunothrombosis, and organ dysfunction in sepsis [16–18]. From this perspective, increasing serum NLRP3 over the first 3 ICU days may reflect unresolved inflammasome-related activity and persistent host-response dysregulation. At the same time, our findings should not be interpreted as proving a direct mechanistic effect, and serum NLRP3 should be regarded as a surrogate biomarker rather than a direct readout of intracellular inflammasome activation. This distinction is clinically relevant because commercial serum NLRP3 assays may differ in antibody specificity, calibration, sample matrix effects, and detection of full-length versus fragmented circulating NLRP3-related proteins. In the absence of internationally recognized reference standards, the ΔNLRP3 threshold observed in this cohort should not be adopted in external settings without local assay validation and recalibration.

Our results should also be interpreted in the context of previous clinical studies. Prior reports have shown that serum NLRP3 is associated with septic shock risk, short-term mortality, and outcomes in sepsis complicated by acute respiratory distress syndrome [19, 20]. In addition, sequential changes in NLRP3 inflammasome-related activation have previously been linked to mortality in sepsis [21]. The present study extends this literature in two ways. First, it explicitly used a prespecified day-3 landmark design to reduce guarantee-time bias when evaluating a change-based biomarker [23–25]. Second, it showed that admission NLRP3 alone had limited standalone discrimination, whereas early serial assessment carried more prognostic information. An important exploratory finding was that day-3 serum NLRP3 alone demonstrated discriminative performance comparable to ΔNLRP3. This observation has practical implications. Calculation of ΔNLRP3 requires paired day-1 and day-3 measurements, whereas a single day-3 reassessment may be simpler to implement in clinical workflows. Therefore, the prognostic signal observed in this study may reflect the patient’s inflammatory status at early reassessment rather than the mathematical change value alone. Nevertheless, day-3 NLRP3 should not be interpreted as an admission biomarker, because it is only available among patients who survive to the landmark time-point. These findings should be considered exploratory and require external validation before day-3 NLRP3 or ΔNLRP3 thresholds are applied in practice. Because ΔNLRP3 is mathematically coupled to both day-1 and day-3 measurements, part of its association may reflect regression to the mean or measurement variability rather than true biological change. The comparable performance of day-3 NLRP3 alone therefore supports a cautious interpretation: the main prognostic signal may lie in early day-3 reassessment, rather than in the change metric itself.

From a clinical standpoint, the most plausible use case for serial serum NLRP3 is not admission triage, but day-3 reassessment among patients who remain in the ICU. In this context, ΔNLRP3 improved discrimination beyond a baseline model including age, sex, admission SOFA score, and log-transformed day-1 lactate, and the association remained robust after additional adjustment for infection site, mechanical ventilation, and CRRT. These findings suggest that serial serum NLRP3 may complement conventional severity assessment rather than replace it. Compared with established dynamic markers such as lactate clearance [9] and procalcitonin kinetics [10], and with investigational inflammatory trajectories summarized in recent biomarker reviews [28], serum NLRP3 remains less standardized and less clinically mature. Therefore, the present findings should be viewed as evidence for potential complementary risk-updating value rather than as evidence that serum NLRP3 outperforms existing dynamic reassessment tools. Direct comparisons with lactate clearance, procalcitonin kinetics, IL-6 trajectories, and changes in SOFA score should be included in future validation studies. The exploratory model including both baseline day-1 NLRP3 and ΔNLRP3 further supports the notion that repeated measurement may contain more information than a single admission value alone. More broadly, these findings align with recent biomarker-development frameworks emphasizing that clinically useful biomarkers require a clearly defined context of use, careful attention to timing, and validation in the target population [28, 29].

Several limitations should be acknowledged. First, this was a retrospective single-center study, and the sample size was modest, particularly for the complete-case primary model, which included 147 patients and 30 post-landmark deaths. Given the inclusion of five independent variables in the primary multivariable logistic regression model, the events-per-variable ratio was 6, which is below the traditional rule-of-thumb of 10. Although bootstrap internal validation supported the stability of the ΔNLRP3-extended model, with an optimism-corrected AUC of 0.802, these results should still be interpreted as derivation-level findings rather than evidence of external generalizability. Second, by design, the day-3 landmark approach excluded patients who died before day 3; therefore, these findings apply to patients who survived to the landmark and should not be extrapolated to very early mortality prediction from ICU admission. Third, serum NLRP3 was measured at only two early time points, which limits inference regarding the full temporal complexity of immune trajectories during sepsis. Fourth, the primary multivariable and ROC analyses used a complete-case approach because day-1 lactate was missing in 14 patients (8.7%). We therefore performed a multiple-imputation sensitivity analysis, which yielded results consistent with the complete-case analysis. However, the imputation model relied on a missing-at-random assumption, and residual selection bias due to unmeasured factors cannot be excluded. Fifth, we did not directly compare serial serum NLRP3 with other dynamic clinical reassessment variables, such as lactate clearance, serial procalcitonin, or changes in SOFA score, which would be important in future work. Finally, assay harmonization and external validation are necessary before any clinical implementation [28, 29].

In summary, this study supports early serial serum NLRP3 assessment as a potentially useful biomarker approach for post-landmark risk updating in sepsis. Rather than supporting admission prediction from a single baseline measurement, the current findings suggest that repeated NLRP3 assessment during the first 3 ICU days may provide additional prognostic information beyond admission clinical severity. External validation studies are needed to determine whether serial serum NLRP3 can contribute to multi-biomarker risk stratification strategies or prognostic enrichment in future sepsis trials.

## Conclusion

In this single-center day-3 landmark cohort of ICU patients with sepsis, early serial serum NLRP3 assessment, summarized as ΔNLRP3, was associated with greater post-day-3 28-day mortality and improved discrimination beyond a baseline clinical model including age, sex, admission SOFA score, and log-transformed day-1 lactate. These findings support further external validation of serial serum NLRP3 as a day-3 risk-updating biomarker for patients who remain in the ICU through day 3, rather than as an admission prediction tool.

## Electronic Supplementary Material

Below is the link to the electronic supplementary material.

Supplementary Material 1

## Data Availability

The datasets used and/or analyzed during the current study are available from the corresponding author on reasonable request.

## Abbreviations

NLRP3: NLR family pyrin domain-containing 3
ΔNLRP3: Change in NLRP3 level from day 1 to day 3
IL-1β: Interleukin-1 beta
IL-18: Interleukin-18
ICU: Intensive Care Unit
SOFA: Sequential Organ Failure Assessment
CRRT: Continuous Renal Replacement Therapy
ROC: Receiver Operating Characteristic
AUC: Area under the curve
CI: Confidence interval
aOR: Adjusted odds ratio
MV: Mechanical ventilation
CRP: C-reactive protein
PCT: Procalcitonin
ELISA: Enzyme-linked immunosorbent assay
MICE: Multiple imputation by chained equations
AIC: Akaike information criterion
LRT: Likelihood-ratio test
