# Supplementary material for: Association of early serial serum NLRP3 measurements with 28-day mortality in sepsis: a single-center retrospective landmark cohort study
Source: BMC Infect Dis. 2026 Jun 1;26:1436. doi: 10.1186/s12879-026-13650-7 (PMC13445758; doi:10.1186/s12879-026-13650-7)
Supplement: Supplementary file 1 [file 12879_2026_13650_MOESM1_ESM.docx]

Table S1. Baseline characteristics of the day-3 landmark cohort stratified by 28-day survival status

| Characteristic | Survivor (n = 128) | Non-survivor (n = 33) | P value |
| --- | --- | --- | --- |
| Age, years | 67.00 (53.00–79.00) | 64.00 (44.00–79.00) | 0.418 |
| Sex, n (%) |  |  | >0.999 |
| Female | 60 (46.9%) | 15 (45.5%) |  |
| Male | 68 (53.1%) | 18 (54.5%) |  |
| SOFA score at day 1 | 6.00 (4.00–7.00) | 8.00 (5.00–10.00) | 0.008 |
| Infection site, n (%) |  |  | 0.864 |
| Lung | 54 (42.2%) | 14 (42.4%) |  |
| Abdomen | 28 (21.9%) | 9 (27.3%) |  |
| Urinary tract | 27 (21.1%) | 5 (15.2%) |  |
| Other | 19 (14.8%) | 5 (15.2%) |  |
| Culture-positive, n (%) |  |  | 0.307 |
| Positive | 91 (71.1%) | 27 (81.8%) |  |
| Negative | 37 (28.9%) | 6 (18.2%) |  |
| Gram type, n (%) |  |  | 0.351 |
| Gram-negative | 48 (37.5%) | 14 (42.4%) |  |
| Gram-positive | 35 (27.3%) | 8 (24.2%) |  |
| Other | 9 (7.0%) | 5 (15.2%) |  |
| Unknown | 36 (28.1%) | 6 (18.2%) |  |
| Any comorbidity, n (%) | 69 (53.9%) | 24 (72.7%) | 0.079 |
| Continuous renal replacement therapy, n (%) | 35 (27.3%) | 16 (48.5%) | 0.034 |
| Mechanical ventilation, n (%) | 59 (46.1%) | 22 (66.7%) | 0.056 |
| NLRP3 at day 1, ng/mL | 3.27 (2.39–4.62) | 2.49 (2.27–3.38) | 0.066 |
| C-reactive protein at day 1, mg/L | 147.46 (93.01–204.60) | 137.82 (114.35–187.31) | 0.895 |
| Procalcitonin at day 1, ng/mL | 7.25 (3.69–11.62) | 7.22 (3.78–20.18) | 0.475 |
| Lactate at day 1, mmol/L | 3.71 (1.89–5.71) | 5.04 (2.22–7.91) | 0.117 |

Note. Continuous variables are presented as median (Q1–Q3), and categorical variables are presented as n (%). P values were calculated using the Wilcoxon rank-sum test, Pearson’s chi-squared test, or Fisher’s exact test, as appropriate. SOFA, Sequential Organ Failure Assessment; NLRP3, NLR family pyrin domain-containing 3.

Table S2. Sensitivity logistic regression analyses

| Variable | + Infection site aOR (95% CI) | P value | + MV aOR (95% CI) | P value | + CRRT aOR (95% CI) | P value |
| --- | --- | --- | --- | --- | --- | --- |
| Age, per year | 0.99 (0.96–1.01) | 0.284 | 0.98 (0.96–1.01) | 0.191 | 0.98 (0.96–1.00) | 0.126 |
| Male sex | 1.07 (0.40–2.88) | 0.890 | 1.18 (0.45–3.18) | 0.741 | 1.18 (0.44–3.22) | 0.748 |
| SOFA score at day 1 | 1.25 (1.01–1.57) | 0.049 | 1.19 (0.96–1.50) | 0.122 | 1.21 (0.97–1.52) | 0.093 |
| Log-transformed day-1 lactate | 1.20 (0.34–4.46) | 0.779 | 1.24 (0.38–4.29) | 0.721 | 1.08 (0.32–3.86) | 0.906 |
| ΔNLRP3, per 1 ng/mL increase | 2.10 (1.52–3.10) | <0.001 | 2.05 (1.50–2.97) | <0.001 | 2.03 (1.48–2.95) | <0.001 |
| MV | — | — | 1.86 (0.62–5.75) | 0.271 | — | — |
| CRRT | — | — | — | — | 2.46 (0.86–7.18) | 0.094 |

Note. Sensitivity analyses were performed by adding infection site, mechanical ventilation (MV), or continuous renal replacement therapy (CRRT) to the primary complete-case logistic model.

Table S3. Exploratory comparison between the primary ΔNLRP3 model and a model including both baseline day-1 NLRP3 and ΔNLRP3

| Variable | Primary ΔNLRP3 model aOR (95% CI) | P value | Baseline day-1 NLRP3 + ΔNLRP3 model aOR (95% CI) | P value |
| --- | --- | --- | --- | --- |
| Age, per year | 0.98 (0.96–1.01) | 0.199 | 0.98 (0.96–1.01) | 0.156 |
| Male sex | 1.11 (0.43–2.96) | 0.829 | 1.08 (0.41–2.93) | 0.872 |
| SOFA score at day 1, per point | 1.24 (1.01–1.55) | 0.045 | 1.17 (0.94–1.46) | 0.159 |
| Log-transformed day-1 lactate | 1.32 (0.41–4.50) | 0.646 | 1.38 (0.42–4.82) | 0.603 |
| Baseline day-1 NLRP3, per 1 ng/mL increase | — | — | 1.98 (1.09–3.90) | 0.035 |
| ΔNLRP3, per 1 ng/mL increase | 2.03 (1.49–2.93) | <0.001 | 3.10 (1.87–5.89) | <0.001 |

Note. Primary ΔNLRP3 model included age, sex, admission SOFA score, log-transformed day-1 lactate, and ΔNLRP3. The exploratory repeated-measurement model additionally included baseline day-1 serum NLRP3. Models were compared using the likelihood ratio test (P = 0.024). Analyses were performed in the complete-case day-3 landmark cohort (n=147; 30 post-landmark deaths). aOR, adjusted odds ratio; SOFA, Sequential Organ Failure Assessment; NLRP3, NLR family pyrin domain-containing 3.

Table S4. Baseline characteristics according to availability of day-1 lactate

| Characteristic | Available day-1 lactate (n = 147) | Missing day-1 lactate (n = 14) | P value |
| --- | --- | --- | --- |
| Age, years | 65.00 (51.50–79.00) | 78.00 (67.75–79.75) | 0.089 |
| Sex, n (%) |  |  | 0.165 |
| Female | 66 (44.9%) | 9 (64.3%) |  |
| Male | 81 (55.1%) | 5 (35.7%) |  |
| SOFA score at day 1 | 6.00 (5.00–8.50) | 5.50 (4.00–6.00) | 0.108 |
| Infection site, n (%) |  |  | 0.614 |
| Lung | 64 (43.5%) | 4 (28.6%) |  |
| Abdomen | 33 (22.4%) | 4 (28.6%) |  |
| Urinary tract | 28 (19.0%) | 4 (28.6%) |  |
| Other | 22 (15.0%) | 2 (14.3%) |  |
| Culture-positive, n (%) | 106 (72.1%) | 12 (85.7%) | 0.356 |
| Gram type, n (%) |  |  | 0.555 |
| Gram-negative | 54 (36.7%) | 8 (57.1%) |  |
| Gram-positive | 40 (27.2%) | 3 (21.4%) |  |
| Other | 13 (8.8%) | 1 (7.1%) |  |
| Unknown | 40 (27.2%) | 2 (14.3%) |  |
| Any comorbidity, n (%) | 84 (57.1%) | 9 (64.3%) | 0.605 |
| Continuous renal replacement therapy, n (%) | 49 (33.3%) | 2 (14.3%) | 0.229 |
| Mechanical ventilation, n (%) | 78 (53.1%) | 3 (21.4%) | 0.024 |
| NLRP3 at day 1, ng/mL | 3.12 (2.36–4.48) | 2.51 (2.22–2.99) | 0.149 |
| NLRP3 at day 3, ng/mL | 3.30 (1.42–4.02) | 3.35 (2.98–3.89) | 0.773 |
| ΔNLRP3, ng/mL | -0.49 (-1.80–0.93) | 0.54 (-1.06–1.22) | 0.198 |
| C-reactive protein at day 1, mg/L | 146.16 (98.65–205.92) | 105.31 (86.48–175.29) | 0.124 |
| Procalcitonin at day 1, ng/mL | 8.17 (3.93–15.20) | 7.25 (4.93–10.04) | 0.583 |
| Post-landmark 28-day mortality, n (%) | 30 (20.4%) | 3 (21.4%) | 1.000 |

Note. Continuous variables are presented as median (Q1–Q3), and categorical variables are presented as n (%). P values were calculated using the Wilcoxon rank-sum test, Pearson’s chi-squared test, or Fisher’s exact test, as appropriate. SOFA, Sequential Organ Failure Assessment; NLRP3, NLR family pyrin domain-containing 3; CRRT, continuous renal replacement therapy.

Table S5. Multiple-imputation sensitivity analysis for post-day-3 28-day mortality

| Variable | Pooled aOR | 95% CI | P value |
| --- | --- | --- | --- |
| Age, per year | 0.99 | 0.96–1.01 | 0.268 |
| Male sex | 1.37 | 0.54–3.47 | 0.506 |
| SOFA score at day 1, per point | 1.25 | 1.02–1.55 | 0.035 |
| Log-transformed day-1 lactate | 1.27 | 0.39–4.13 | 0.684 |
| ΔNLRP3, per 1 ng/mL increase | 2.15 | 1.53–3.02 | <0.001 |

Note. Multiple imputation by chained equations was used to impute missing day-1 lactate values in the full day-3 landmark cohort (N=161). Fifty imputed datasets were generated using predictive mean matching for day-1 lactate, and estimates were pooled using Rubin’s rules. The model included age, sex, admission SOFA score, log-transformed day-1 lactate, and ΔNLRP3. aOR, adjusted odds ratio; SOFA, Sequential Organ Failure Assessment; NLRP3, NLR family pyrin domain-containing 3.

Table S6. Bootstrap internal validation of clinical prediction models

| Model | Apparent AUC (95% CI) | AUC optimism | Optimism-corrected AUC (95% CI) | Apparent Brier score | Brier optimism | Optimism-corrected Brier score (95% CI) |
| --- | --- | --- | --- | --- | --- | --- |
| Baseline clinical model | 0.669 (0.553–0.785) | 0.053 | 0.616 (0.512–0.716) | 0.149 | 0.012 | 0.161 (0.127–0.200) |
| Baseline clinical model + day-3 NLRP3 | 0.814 (0.725–0.903) | 0.033 | 0.781 (0.702–0.865) | 0.123 | 0.014 | 0.137 (0.105–0.175) |
| Baseline clinical model + ΔNLRP3 | 0.828 (0.727–0.930) | 0.027 | 0.802 (0.715–0.895) | 0.111 | 0.013 | 0.123 (0.091–0.159) |

Note. Bootstrap internal validation was performed using 1,000 resamples in the complete-case day-3 landmark cohort (n=147; 30 deaths). The baseline clinical model included age, sex, admission SOFA score, and log-transformed day-1 lactate. Optimism was estimated by comparing bootstrap-sample and test-sample performance. Lower Brier scores indicate better overall prediction accuracy. SOFA, Sequential Organ Failure Assessment; NLRP3, NLR family pyrin domain-containing 3; AUC, area under the receiver operating characteristic curve.

Table S7. Exploratory comparison of clinical models incorporating day-1 NLRP3, day-3 NLRP3, and ΔNLRP3

| Model | AUC (95% CI) | Brier score | AIC | LRT vs. baseline clinical model, P value | LRT: full two-time-point model vs. reduced model, P value |
| --- | --- | --- | --- | --- | --- |
| Baseline clinical model | 0.669 (0.553–0.785) | 0.149 | 148.68 | — | <0.001 |
| Baseline clinical model + day-1 NLRP3 | 0.707 (0.594–0.820) | 0.141 | 145.64 | 0.025 | <0.001 |
| Baseline clinical model + day-3 NLRP3 | 0.814 (0.725–0.903) | 0.123 | 128.04 | <0.001 | 0.0068 |
| Baseline clinical model + ΔNLRP3 | 0.828 (0.727–0.930) | 0.111 | 125.79 | <0.001 | 0.0245 |
| Baseline clinical model + day-1 NLRP3 + day-3 NLRP3 | 0.842 (0.751–0.934) | 0.108 | 122.73 | <0.001 | — |

Note. All models included age, sex, admission SOFA score, and log-transformed day-1 lactate as baseline clinical covariates. The full two-time-point model additionally included both day-1 and day-3 serum NLRP3. The column “LRT vs. baseline clinical model” evaluates improvement after adding the listed NLRP3-related measure(s) to the baseline clinical model. The column “LRT: full two-time-point model vs. reduced model” evaluates whether the full two-time-point model improved fit compared with the corresponding reduced model in that row. Because the day-3 NLRP3 model and the ΔNLRP3 model are not nested, they were directly compared using DeLong’s test rather than likelihood-ratio testing; this comparison showed no significant difference in discrimination (AUC, 0.814 vs. 0.828; DeLong P=0.712). Analyses were performed in the complete-case day-3 landmark cohort (n=147; 30 post-landmark deaths). SOFA, Sequential Organ Failure Assessment; NLRP3, NLR family pyrin domain-containing 3; AUC, area under the receiver operating characteristic curve; AIC, Akaike information criterion; LRT, likelihood-ratio test.
